# Network pharmacology- and molecular docking-based investigation on the mechanism of action of Si-ni San in the treatment of depression combined with anxiety and experimental verification in adolescent rats

**DOI:** 10.3389/fpsyt.2024.1414242

**Published:** 2024-08-23

**Authors:** Zhiping Li, Shimin Liang, Xulan Cui, Chongkun Shen, Zaibin Xu, Wei Chen, Mingan Wu, Chao Liang, Jinman Liu, Jiawen Huang, Weirong Li

**Affiliations:** ^1^ Science and Technology Innovation Center, Guangzhou University of Chinese Medicine, Guangzhou, China; ^2^ Clinical Medical College of Acupuncture Moxibustion and Rehabilitation, Guangzhou University of Chinese Medicine, Guangzhou, China; ^3^ Department of Pharmacy, Shenzhen Hospital, Southern Medical University, Shenzhen, China; ^4^ School of Pharmaceutical Sciences, Guangzhou University of Chinese Medicine, Guangzhou, China; ^5^ School of Fundamental Medical Science, Guangzhou University of Chinese Medicine, Guangzhou, China; ^6^ Rehabilitation Center Massage Clinic, The First Affiliated Hospital of Guangzhou University of Chinese Medicine, Guangzhou, China; ^7^ Deparment of Acupuncture, Haikou Hospital of Traditional Chinese Medicine, Haikou, China; ^8^ Department of Encephalopathy, Affiliated Jiangmen Traditional Chinese Medicine (TCM) Hospital of Ji'nan University, Jiangmen, China

**Keywords:** network pharmacology, molecular docking, Si-ni San, depression, anxiety, maternal separation, chronic unpredictable mild stress

## Abstract

**Background:**

The incidence rate of adolescent depression and anxiety has been increasing since the outbreak of COVID-19, which there are no effective therapeutic drugs available. Si-ni San is commonly used in traditional Chinese medicine for the treatment of depression-like as well as anxiety-like behavior, but its mechanism for treating depression combined with anxiety during adolescence is not yet clear.

**Methods:**

Network pharmacology was used to explore potential drug molecules and related targets, molecular docking and molecular dynamics (MD) simulation were used to evaluate the interaction between the potential drug molecules and related targets, and a model of anxiety combined with depression in adolescent rats as well as the following behavioral tests and molecular biology tests were used to verify the results from network pharmacology and molecular docking.

**Results:**

As a result, 256 active ingredients of Si-ni San and 1128 potential targets were screened out. Among them, quercetin, Luteolin, kaempferol, 7-Methoxy-2-methyl isoflavone, formononetin showed to be the most potential ingredients; while STAT3, IL6, TNF, AKT1, AKT1, TP53, IL1B, MAPK3, VEGFA, CASP3, MMP9 showed to be the most potential targets. AGE-RAGE signaling pathway in diabetic complications, IL-17 signaling pathway, HIF-1 signaling pathway, PI3K-Akt signaling pathway and TNF signaling pathway, which are involved in anti-inflammation processes, showed to be the most probable pathways regulated by Si-ni San. Molecular docking and MD simulation between the compounds to inflammation-associated targets revealed good binding abilities of quercetin, Luteolin, kaempferol, nobiletin and formononetin to PTGS2 and PPARγ. In the experiment with adolescent rats, Si-ni San markedly suppressed early maternal separation (MS) combined with adolescent chronic unpredictable mild stress (CUMS)-induced depression combined with anxiety. The qPCR results further indicated that Si-ni San regulated the oxidative stress and inflammatory response.

**Conclusion:**

This study demonstrates that adolescent anxiety- and depression-like behavior induced by MS combined CUMS can be ameliorated by Si-ni San by improved inflammation in hippocampus via targeting TNF pathway and Nrf2 pathway, helping to reveal the mechanism of Si-ni San in treating adolescent depression combined with anxiety.

## 1 Introduction

Depression, as a common mental health issue, has shown an increasing trend in its epidemiology worldwide among the elderly and young (1, 2). In the past 10 years, the incidence rate of depression in adolescent showed a significant upward trend due to long-term isolation, reduced social activities, increased learning pressure, accelerated social rhythm and increased life pressure (3). In addition, anxiety, as a common complication of depression, which not only exacerbates the symptoms of depression, but may also lead to physiological reactions such as insomnia, palpitations, and shortness of breath, seriously affecting their daily life and study (4). Therefore, intervention is needed before the formation of depression and anxiety in adolescents to reduce the incidence rate.

The pathogenesis of depression combined with anxiety is quite complex, involving multiple links and pathways, and its specific regulatory network is currently not fully understood, including reduction of synaptic density, evening circadian preference, microbial changes mediated host metabolic disorders as well as neuroinflammation and cell apoptosis (5–8). Study has confirmed that congenital and adaptive immune system dysregulation occur in patients with depression, which can hinder good prognosis as well as response to antidepressants (9). Also, inflammation may also be a key disease regulator, promoting susceptibility to depression. High cholesterol diet intake promotes depression and anxiety like behavior in mice through gut microbiota by causing neuroinflammatory disorders (10). Therefore, regardless of whether inflammation is secondary to early life trauma, acute stress response, changes in microbiota, genetic susceptibility, controlling inflammation may provide overall therapeutic efficacy (9).

Traditional Chinese medicine has shown unique advantages in the treatment of depression, with its multi-component and multi-target characteristics comprehensively regulating multiple systems of the body (11). As a classic formula in traditional Chinese medicine, Si-ni San is commonly used in the treatment of clinical depression, its specific material basis and mechanism of action are still not fully understood. Our previously discovered that Si-ni San can enhance synaptic plasticity through the CaSR-PKC-ERK signaling pathway, thereby improving depressive like behavior in rats (12). Meanwhile, it can also improve depressive like behavior in maternal isolated rats subjected to early stress through the 5-HT1A receptor/CREB/BDNF pathway (13). However, further exploration is needed on the deeper mechanisms and active molecules of Si-ni San.

Current study used a depression combined with anxiety model in adolescent rats under dual stimulation of early maternal separation (MS) and chronic unpredictable mild stress (CUMS), to explore the efficacy and mechanism of Si-ni San on anxiety combined with depression like behavior in adolescent rats. This is the dual stimulus model established in our previous studies, and we found that both female and male adolescent rats in this model exhibit depressive and anxiety like behaviors, which may be related to changes in synaptic plasticity (14, 15). Meanwhile, we used network pharmacology and molecular docking to explore the main components and molecular biological mechanisms of Si-ni San in treating depression combined with anxiety, providing relevant references for subsequent research.

## 2 Materials and methods

### 2.1 Establishment of the Si-ni San-ingredient-target interaction

The Traditional Chinese Medicine Systems Pharmacology Database and Analysis Platform (TCMSP, https://old.tcmsp-e.com/tcmsp.php) were used to collect the chemical components in Si-ni San (“Chaihu”, “Bai Shao”, “Zhi Shi”, and “Gan Cao”), including Mol ID, Molecule Name, MW, OB (%), DL information, etc. Retrieve genes related to anxiety and depression using the keywords “anxiety” and “depression” in the Disgnet database, GeneCards database, Malacard database, and TTD database, and the common targets were selected. Through the Draw Venn Diagram website (http://bioinfogp.cnb.csic.es/tools/venny/index.html), the potential targets of Si-ni San and the disease targets of depression combined with anxiety were mapped, and the common targets of Si-ni San and anxiety related depression were screen out as potential targets.

### 2.2 Construction of a protein–protein interaction network

Upload the screened potential targets of Si-ni San for treating depression combined with anxiety to the online website STRING 11.5 (https://string-db.org) (16) and the relationship between protein interactions were obtained then visualized using Cytoscape 3.8.2 software (17). Meanwhile, using the CytoHubba plugin to screen by Degree, Betweenness as well as Closeness, the top 10 proteins (sort by degree value) with the strongest interaction were obtained.

### 2.3 Gene ontology enrichment and pathway analysis

Gene ontology (GO) analysis (biological processes, molecular functions, and cellular components) and Kyoto Encyclopedia of Genes and Genomes (KEGG) pathway analysis were performed on core genes using the Metascape database (18). By inputting the name of the target gene and setting a threshold P value of<0.05, the online mapping website (http://www.bioinformatics.com.cn) (19) was used to screen the top 20 biological processes in GO analysis and the top 20 pathways in KEGG analysis, and visualize the results.

### 2.4 Molecular docking

Two- and three- dimensional structural information of Si-ni San active ingredients were obtained from the PubChem database (https://pubchem.ncbi.nlm.nih.gov/) (20), and Chem Bio 3D software was performed to optimize structure by energy minimization means, and the RCSB protein data bank (https://www.rcsb.org/) (21) was used to collected all the crystal structures of the targets (PTGS2 (PDBID:5f19); ESR1 (PDBID:6iar); NOS2 (PDBID: 3e7g); GSK3B (PDBID:1j1b) and PPARG (PDBID:3et3). Molecular docking was carried out using LeDock software (22) and BIOVID Discovery studio 2019 Client. Proteins preparing was performed using Pymol software (including delete water and ligand, add hydrogen and Lepro plugin was performed to add charge). Molecular docking was performed by LeDock software to obtain the binding energy score, and displayed in the form of heatmap. The Discovery 2019 client software was used to visualize the interaction forces between the optimal target protein and its corresponding core components.

### 2.5 Molecular dynamics simulation

Gromacs 2020 software was used for MD simulation and combined free energy calculation, as same as our previous study (23). The AMBER99SB-ILDN force field parameters and gaff2 universal force field parameters were used. The Sobtop program was used to construct the compound topology, and RESP was used for charge fitting. The TIP3P dominant water model was selected, where the minimum distance between atoms in the protein and the edge of the water box was 1.0 nm. Sodium or chloride ions was used to neutralize the system charge based on the docking results. The workflow of MD simulation included energy minimization, heating, equilibrium, production dynamics simulation, etc. Firstly, the heavy atoms of proteins (and compounds) were constrained and the energy of water molecules were minimized through 10000 steps (including 5000 steps of steepest descent and 5000 steps of conjugate gradient); then the constraints were released and 10000 steps of energy minimization were performed on the entire system (including 5000 steps of steepest descent method and 5000 steps of conjugate gradient method). After energy optimization, the system was slowly heated to 300 K within 50 ps time; then, the system was equilibrated for 50ps in the npt ensemble. Finally, the system was subjected to a 100ns MD simulation under the npt ensemble, with trajectory data saved every 10 ps and analyzed using the trjconv module.

### 2.6 Animals

Male and female SD rats were obtained from the Center of Animal Experimental of Guangzhou University of Chinese Medicine and housed raised in an SPF level environment (22 °C, 12 h/12 h dark/lights cycle) with free access to food and water in Shenzhen Hospital of Southern Medical University. Offspring male rats were divided into 6 groups: Control (saline), Model (MS+CUMS, saline), Positive (MS+CUMS, 0.5 g/mL fluoxetine), SNS-L (MS+CUMS, 0.25 g/mL Si-ni San), SNS-M (MS+CUMS, 0.5 g/mL Si-ni San) and SNS-H (MS+CUMS, 1 g/mL Si-ni San) as in our previous study (12). Each rat was given different drugs by gavage according to their groups once a day during CUMS, with a dose of 1mL/100g body weight. All the experiments were approved by Ethics Committee of SHSMU on Laboratory Animal Care (No.2024-004).

### 2.7 MS procedure

Male and female rats were mated to produce litter, and the day of birth were defined as postnatal day 0 (PND0). Offspring male rats were selected and grouped at PND0, 8 rats per group. Rats were received MS during PND0 to PND21 except for the rats in Control group. The separation lasted for 6 h per day, which was 09:00-12:00 in the morning and 14:00-17:00 in the afternoon. When rats were received MS, the cages were filled with cotton to maintain the body temperature of rats. At all times outside 6 h of the MS, the cubs stayed with their mothers, until PND21, the rats were separated from their mothers.

### 2.8 CUMS procedure

In brief, rats were exposed to one stressor each day from PND28 to PND56, and the same stressor were not scheduled in 3 consecutive days. The stressors included in this study were as followed: 1) fasting for 24 h; 2) water deprivation for 24 h; 3) cage tilted at 45° for 24 h; 4) wet environment for 24 h; 5) cold water bath for 5 min at 4°C; 6) hot water bath for 5 min at 45°C; 7) crowding with 12 rats in a cage for 24 h; 8) light/dark cycle inversion for 24 h.

### 2.9 Si-ni San preparation

Radix Bupleuri (ChaiHu), Paeonae Alba Radix (Bai-Shao), Aurantii Immaturus Fructus (ZhiShi), and Licorice Root (Gan-Cao) were obtained from Shenzhen Hospital of Southern Medical University with a ratio of 1:1:1:1. All herbs were soaked in 10 times the volume of water and then were heated for 1h. After filtered, the decoction was concentrated to 1 g/ml by a rotary evaporator as in our previous study (12).

### 2.10 Open-field test

A black square arena (100cm ×100cm × 40cm) with a black floor was used for the OFT. Each rat was allowed to freely explore the field for 3 min, and a video tracking system was used to record the total distance and time spent in the central area. After observing one rat, disinfect the arena with 75% ethanol to prevent odor and feces from interfering with the behavior of the next rat.

### 2.11 Sucrose preference test

On the first day, rats were exposed to two bottles of 1% sucrose, and on the second day, one bottle of 1% sucrose was replaced with water. On the third day, the rats were fasted from food and water, and the fourth day, rats were exposed to a bottle of water and a bottle of 1% sucrose solution, and the consumption of water and 1% sucrose were recorded, respectively. Sugar preference=sugar consumption/(water consumption+sugar consumption)×100.

### 2.12 Force swimming test

The rats were placed one by one in a cylindrical glass instrument with a diameter of 20 cm and a water depth of 30 cm, and the water temperature was maintained at 23 ± 1 °C. On the first day, each rat underwent a 15 min adaptation in the instrument. The next day, each rat was received 5 min test in the instrument. The resting time of each rat, which defined as maintaining balance with no movement or only slight limb movements, was recorded using a video tracking system.

### 2.13 Immunohistochemistry

After fixation, the embedded brain were cut into 5-μm slices, then antigen repair was performed using sodium citrate buffer after dewaxing. Slices were incubated with NF-κB p65 antibody (ImmunoWay, USA) overnight at 4 °C and then were incubated with DAB kit as well as secondary antibody. Jiangfeng Pathology Instrument was used to obtain images.

### 2.14 qPCR

Hippocampus tissues were collected and total mRNA of each sample were extracted. After reverse transcription into cDNA using PrimeScript™ RT Master Mix (Takara Biomedical Technology (Beijing) Co., Ltd., China), amplification of cDNA using specific primers was conducted using TB Green® Premix Ex Taq™ II (Takara Biomedical Technology (Beijing) Co., Ltd., China). The specific primers were shown in **Table 1**. β-actin was used as an internal control, and the data from each group was normalized by data from Control group.

**Table 1:** Primers used for the qPCR analysis.

Gene name	Sequence 5′ − 3′ (Forward Primer)	Sequence 5′ − 3′ (Reverse Primer)
*Nrf2*	TGACAATGAGGTTTCTTCGGCTACG	GGAGAGGATGCTGCTGAAGGAATC
*Ho1*	AGAGGCTAAGACCGCCTTCC	AGCGGTGTCTGGGATGAACT
*Stat3*	GACATTCCCAAGGAGGAGGC	CTACCTGGGTCAGCTTCAGG
*Tnf*	CGTCAGCCGATTTGCCATTT	TCCCTCAGGGGTGTCCTTAG
*Cas9*	CTTCCTCGCTTCATCTCCTGCTTAG	GCCTGGGTGTTTCTGGTGTGAG
*Keap1*	TGGTCGCCCTGTGCCTCTATG	TCATCCGCCACTCATTCCTCTCC
*Il18*	TTGGAATCAGACCACTTTGGCAGAC	GTCACAGCCAGTCCTCTTACTTCAC
*β-actin*	CTGTGTTGTCCCTGTATGCCTCTG	GGAACCGCTCATTGCCGATAGTG

### 2.15 Analysis

Data were presented as mean ± standard error of the mean (S.E.M). Data analyses were conducted using GraphPad Prism 6.0 software (San Diego, CA, USA). In brief, mean values were compared using one-way analysis of variance (ANOVA) and *p* < 0.05 were considered statistically significant.

## 3 Results

### 3.1 Construction of ingredients-targets-related network of Si-ni San

**Figure 1:**
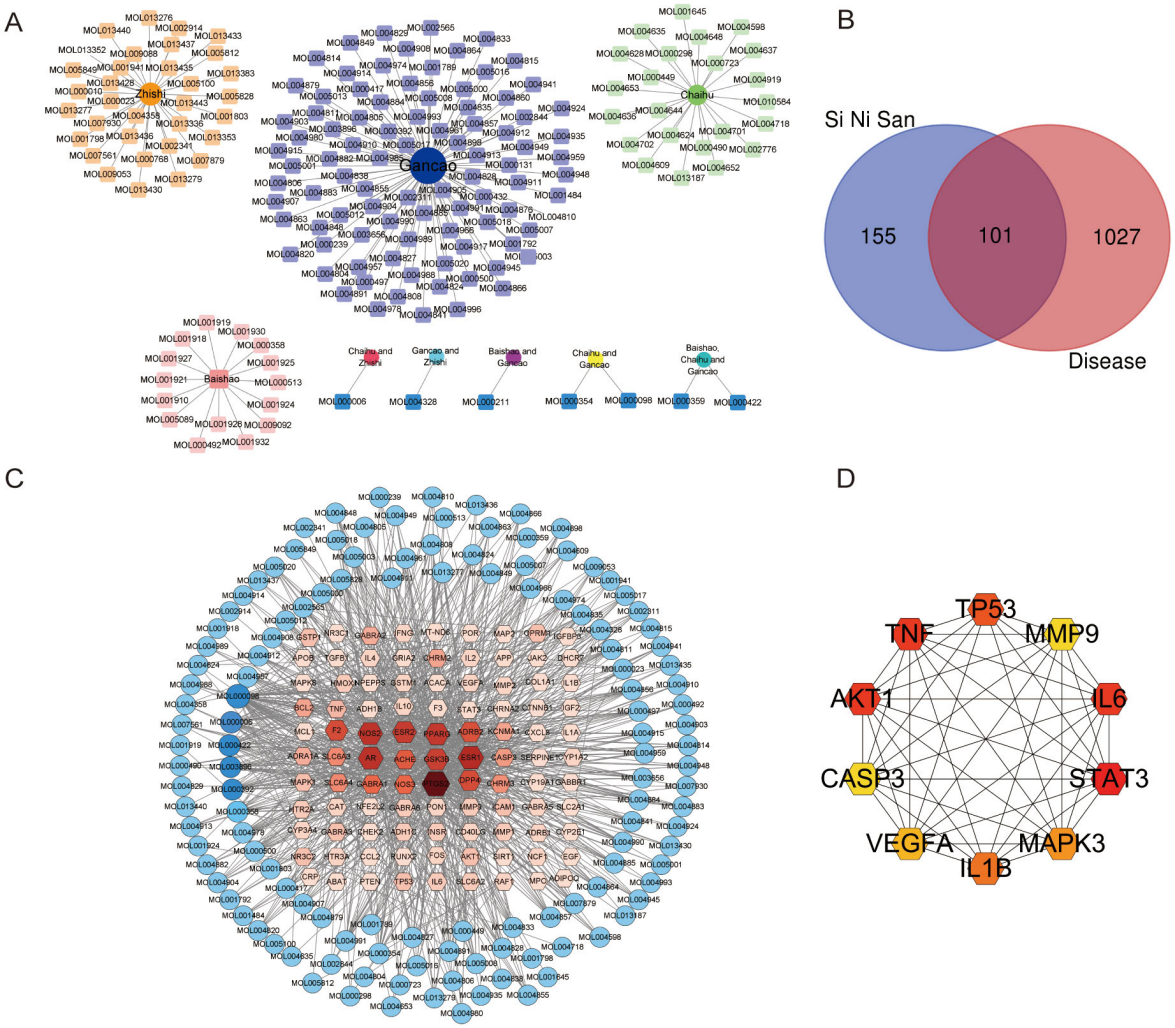
Network pharmacology predicted the active ingredients-target interactions for Si-ni San against depression combined with anxiety. **(A)** The network of herbs and ingredients (Each herb is at the center of each circle, and their components are around them). **(B)** The Venn diagram for the target from ingredients of Si-ni San as well as the targets from disease (depression combined with anxiety). **(C)** Network of targets predicted via the Si-ni San-active ingredients (the red nodes represent the predicted targets while the blue nodes represent the active ingredients in Si-ni San). **(D)** The key targets from top 10 of PPI network (The redder the color, the higher the ranking).

Through the TCMSP database and the CNKI database, the chemical components of 4 traditional Chinese medicines contained in the formula were searched separately. After screening based on OB (%) ≥ 30% and DL ≥ 0.18, and study found that some important ingredients, a total of 170 active ingredients were obtained (**Supplementary Table 2**), including 18 in *Paeonia lactiflora Pall*., 28 in *Bupleurum chinensis DC.*, 97 in *Glycyrrhiza uralensis Fisch.*, and 36 in *Citrus junos Sieb. ex Tanaka* (including 7 common compounds) (**Figure 1A**). A total of 256 potential targets corresponding to the active ingredients were collected through the TCMSP database, and a total of 1128 depression combined with anxiety targets were collected from Disease databases Genecard, TTD, Disgnet, and Malacard. After the construction of the Venn diagram, 101 common targets were selected as the key targets (**Figure 1B**), and a network with 228 nodes and 930 edges was constructed (**Figure 1C**). After uploaded 101 potential targets to the STRING website, a network with the top 10 nodes based on degree value was visualized using Cytoscape 3.8.2 software (**Figure 1D**). The top 10 nodes were as followed: STAT3 (degree=38), IL6 (degree=37), TNF (degree=37), AKT1 (degree=37), TP53 (degree=32), IL1B (degree=31), MAPK3 (degree=29), VEGFA (degree=27), CASP3 (degree=24), MMP9 (degree=24).

### 3.2 Biomedical functional enrichment analysis

**Figure 2:**
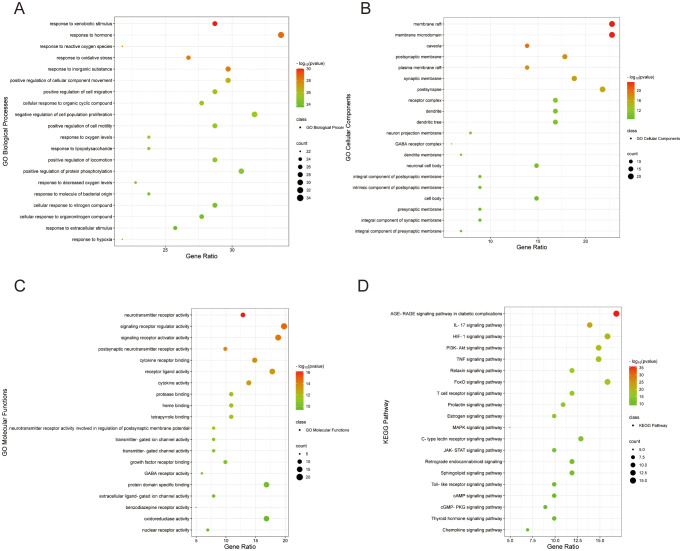
Go and KEGG pathway analysis of the targets of Si-ni San against depression combined with anxiety. **(A)** Biological processes. **(B)** Cellular component. **(C)** Molecular functions. **(D)** KEGG pathway.

The 101 intersecting targets related to Si-ni San on treating anxiety combined with depression were analyzed by GO functional analysis. The top 20 enriched terms including biological process, molecular function and cellular component were shown in **Figures 2A–C** respectively. Basically, the main terms of biological processes were related to xenobiotic stimulus, hormones, reactive oxygen species, oxidative stress, inorganic substance, etc. (**Figure 2A**). The main terms of molecular functions were related to neurotransmitter receptor activity, signaling receptor regulator and activator activity, postsynaptic neurotransmitter receptor activity, cytokine receptor binding, receptor-ligand activity, cytokine activity, etc. (**Figure 2B**). The main terms of cellular components were related to membrane raft, membrane microdomain, caveola, postsynaptic membrane, plasma membrane raft, etc. (**Figure 2C**). Meanwhile, the top 20 terms based on the log*P* value observed from KEGG pathway enrichment were as followed: AGE-RAGE signaling pathway in diabetic complications, IL-17 signaling pathway, HIF-1 signaling pathway, PI3K-Akt signaling pathway, TNF signaling pathway, Relaxin signaling pathway, etc. (**Figure 2D**), which were closely related to inflammatory response.

### 3.3 Interactions between potential targets and ingredients

**Figure 3:**
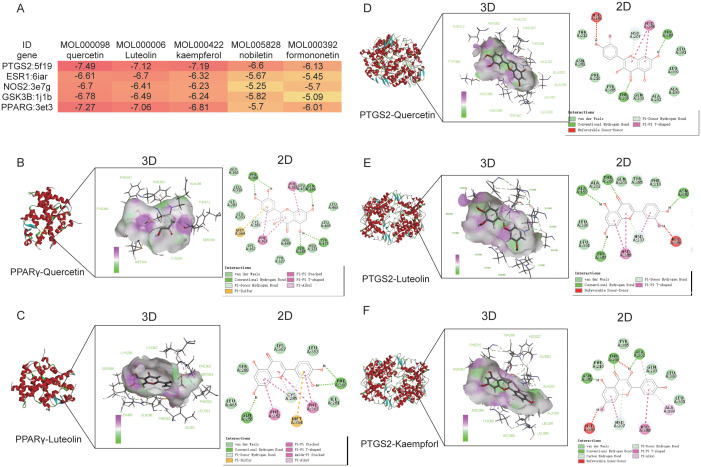
Molecular docking analysis of top 5 active ingredients and top 5 targets. **(A)** Heat Map of molecular docking energies. **(B)** Molecular docking of quercetin binding to PPARγ. **(C)** Molecular docking of Luteolin binding to PPARγ. **(D)** Molecular docking of quercetin binding to PTGS2. **(E)** Molecular docking of Luteolin binding to PTGS2. **(F)** Molecular docking of kaempferol binding to PTGS2.

To gain a more comprehensive understanding of the potential effects of drug components and disease targets, the top 5 components obtained from the network topology of active compound-target- pathway network graph (**Supplementary Table 2**), including quercetin, luteolin, kaempferol, nobiletin and formononetin were each associated with the top 5 targets, including PTGS2, ESR1, NOS2, GSK3B and PPARG. Molecular docking energies were shown in **Figure 3A**, and the energies less than -5 kcal/mol considered as a good combination between target and component. Top 5 ligand-receptor interaction diagram were shown in **Figures 3B–F**. The results showed that the binding energy of quercetin, luteolin binding to PPARG (PDB ID:3et3) were -7.27 kcal/mol (**Figure 3B**) and -7.06 kcal/mol (**Figure 3C**), respectively. The binding energy of luteolin (**Figure 3D**), quercetin (**Figure 3E**), and kaempferol (**Figure 3F**) binding to PTGS2 (PDB ID: 5f1q) were -7.12 kcal/mol, -7.49 kcal/mol and -7.19 kcal/mol, respectively. Hydrogen bond force and van der Waals force are the main force between Si-ni San active ingredients and targets (**Supplementary Table 3**). Our results indicated that quercetin, luteolin, kaempferol, nobiletin and formononetin were capable of binding directly interacting with critical targets to exert their anti-depression and anti-anxiety pharmacological effects.

### 3.4 The dynamics stability simulation of active ingredients with targets

**Figure 4:**
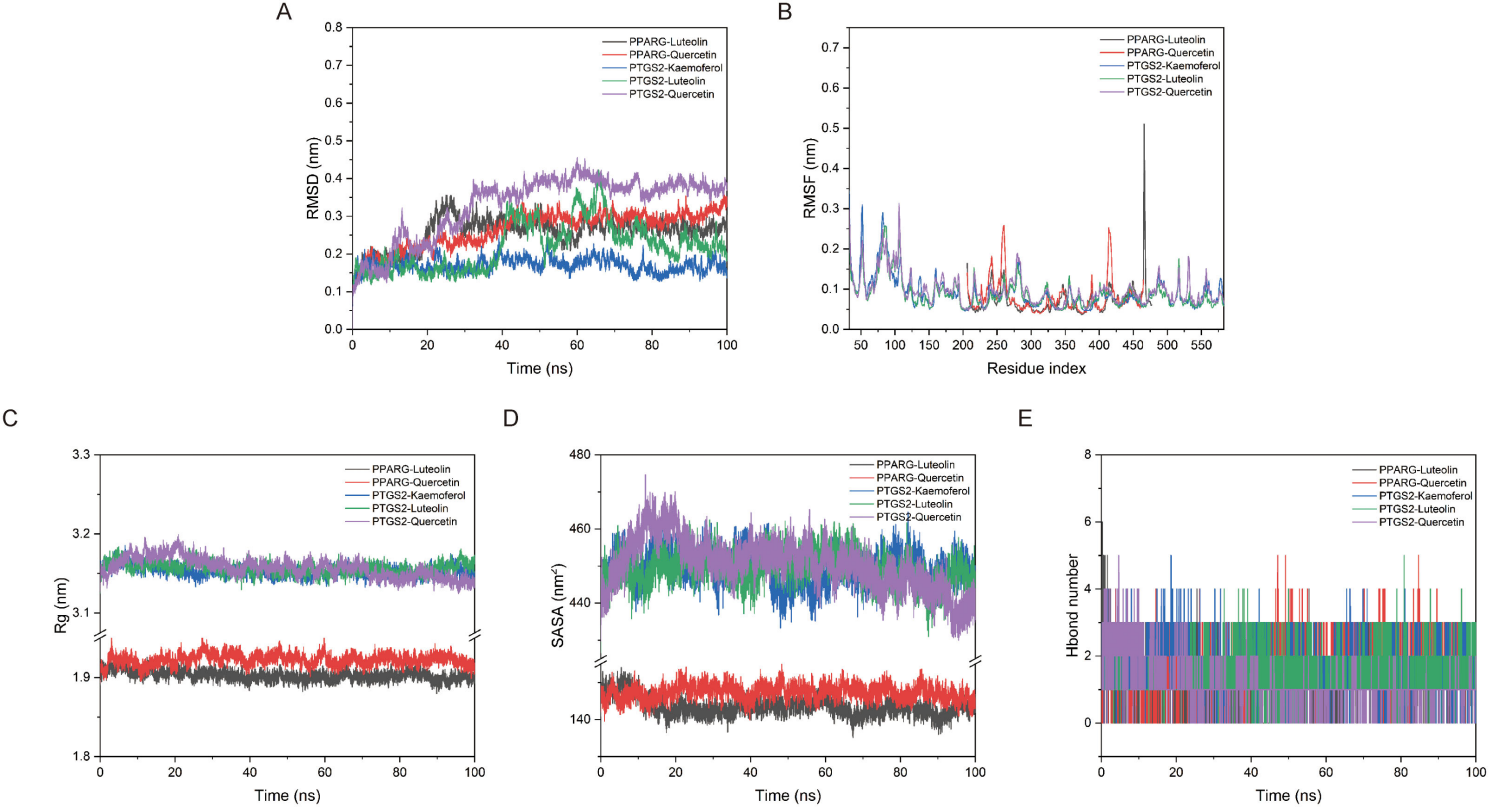
Molecular dynamics simulations confirmed the stability of complexes. **(A)** The RMSD of complexes. **(B)** The RMSF of complexes. **(C)** The SASA of complexes. **(D)** The Rg of complexes. **(E)** The hydrogen bond number of ligand with protein.

To further investigate the interaction between compounds and proteins, a 100 ns molecular dynamics simulation of protein compound complexes were conducted. As shown in **Figure 4A**, the average RMSD of PPARG-Luteolin, PPARG-Quercetin, PTGS2-Kaemoferol, PTGS2-Luteolin, and PTGS2-Quercetin complexes were all less than 4 Å, and the complexes reached dynamic equilibrium at around 40ns. The detailed diagram (**Supplementary Figure 1**) of the interaction between small molecules and proteins also showed that small molecules formed hydrogen bonds and hydrophobic interactions with proteins. According to the RMSF diagram (**Figure 4B**), a small number of amino acids in the complex formed by the interaction between protein and compound underwent significant conformational changes. The main reason was that the amino acids in this part of the complex were located in the hinge region of the protein, which had slightly greater flexibility and was prone to undergo certain conformational changes during the simulation process. As shown in **Figure 4C**, the Rg of the five complex proteins had a decreased to a certain extent during the simulation process which may due to the molecular dynamics, where the binding between the proteins and the compounds promoted the proteins to maintain more hydrophobic contact, resulting in more effective interactions within the proteins to better match the compounds and promote the stability of the complexes. According to **Figure 4D**, it can be seen that the Solvent accessible surface area (SASA) changes of the five complexes had been reduced to a certain extent, and the binding of small molecules to the protein had not disrupted the stability of the protein itself. Based on the hydrogen bond network diagram of each protein and compound, PPARG-Luteolin, PPARG-Quercetin, PTGS2-Kaemoferol, and PTGS2-Quercetin complexes were observed at least one hydrogen bond interaction with protein pocket amino acids, which plays an important role in stable compound protein binding (**Figure 4E**). The binding free energies of PPARG-Luteolin, PPARG-Quercetin, PTGS2-Kaemoferol, PTGS2-Luteolin, and PTGS2-Quercetin proteins to compounds are -77.673 ± 9.21 kJ/mol, -54.688 ± 9.36 kJ/mol, -57.733 ± 8.967 kJ/mol, -93.708 ± 9.953 kJ/mol, and -62.039 ± 7.464 kJ/mol, respectively (**Supplementary Table 4**). These results indicated that the compounds could stably remain in the protein site pocket and had strong van der Waals force interactions with surrounding residues.

### 3.5 Si-ni San blocked MS+CUMS induced depression combined with anxiety in rats

**Figure 5:**
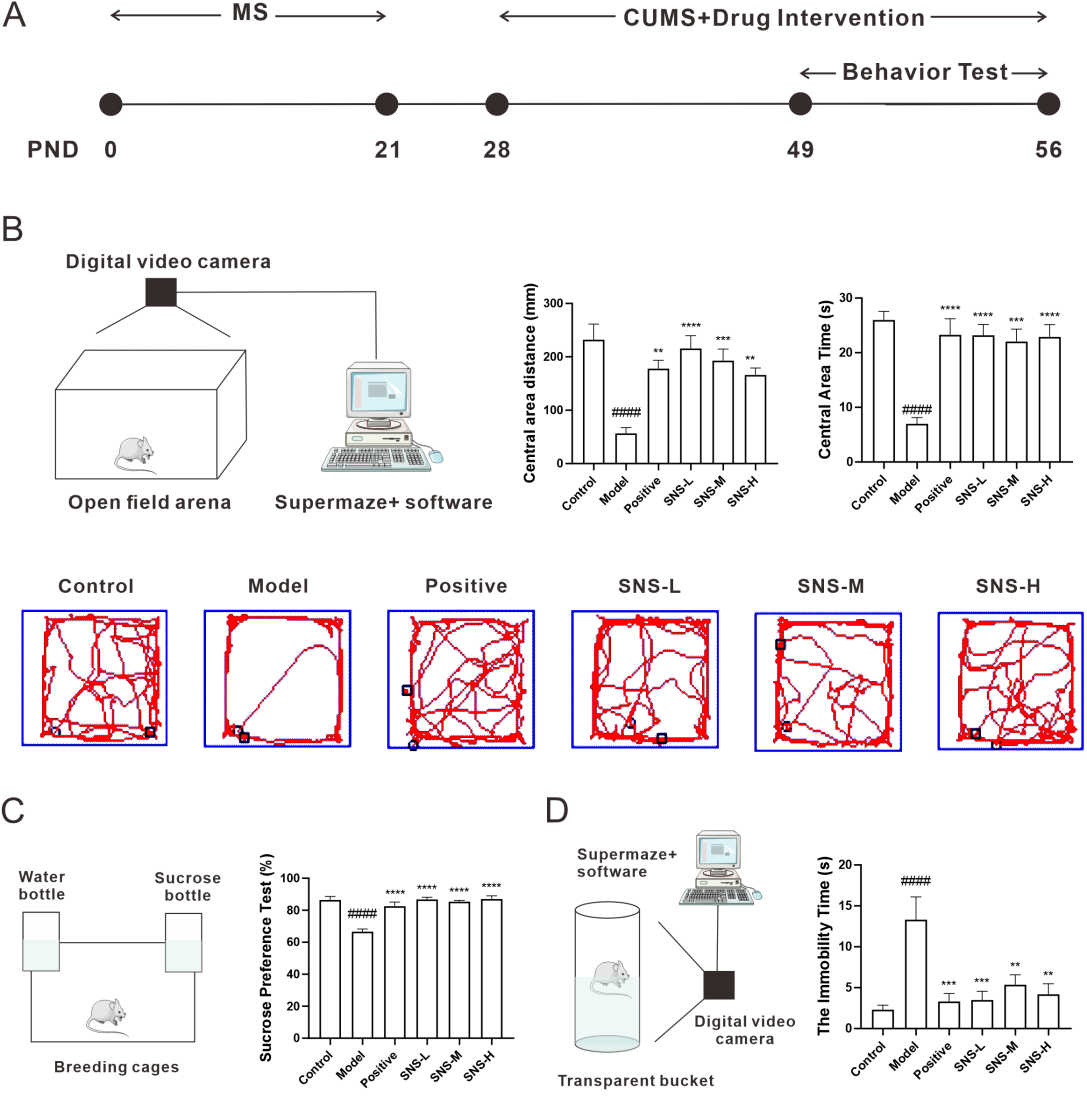
Effect of Si-ni San on behavior tests in stress-exposed rats. **(A)** Schematic diagram of the experiment. **(B)** Open-field test (OFT), schematic diagram of OFT (left), central area distance (middle), central area time (right), and representative diagram of action path (down). **(C)** Sucrose preference test (SPT), schematic diagram of SPT (left), and sucrose preference rate (right). **(D)** Force swimming test (FST), schematic diagram of FST (left), and the immobility time (right). Data were expressed as mean ± S.E.M, n=6. ^####^ *P*<0.001 vs. Control group; ^**^ *P*<0.01, ^***^ *P*<0.005, ^****^ *P*<0.001 vs. Model group.

In order to verify the results of network pharmacology, a model of anxiety combined with depression was established in rats undergoing MS and CUMS, as in our previous study (12). Briefly, rats were subjected to MS from PND0 to PND21, and subjected to CUMS and drug intervention simultaneously from PND28 to PND56, and then OFT, SPT and FST were performed sequentially from PND49 to PND56 to observe the behavior of rats (**Figure 5A**). As shown in **Figure 5A**, rats were placed in an open field arena to observe their autonomous activity, tension, and exploratory behavior. Rats in model group presented a short distance and time in central area while those were increased with Si-ni San invention, which were the similar result as in positive group but it was not dose-dependent (**Figure 5B**). Then, SPT were conducted to measure anhedonia in rats. As expected, rats in model group showed significant anhedonia while those was increased in SNS-L, SNS-M and SNS-H groups as well as positive group (**Figure 5C**). Finally, FST, as the gold index evaluated the therapeutic effect of medicine, was performed to observe the depression- and anxiety-like behavior in rats. Notably, rats in model group showed a high immobility time while those in drug intervention groups were decreased (**Figure 5D**). Our results indicated that Si-ni San ameliorated the depression- and anxiety-like behavior induced by MS+CUMS in adolescent rats.

### 3.6 Si-ni San regulated oxidative stress- and inflammatory response-related genes in hippocampus

**Figure 6:**
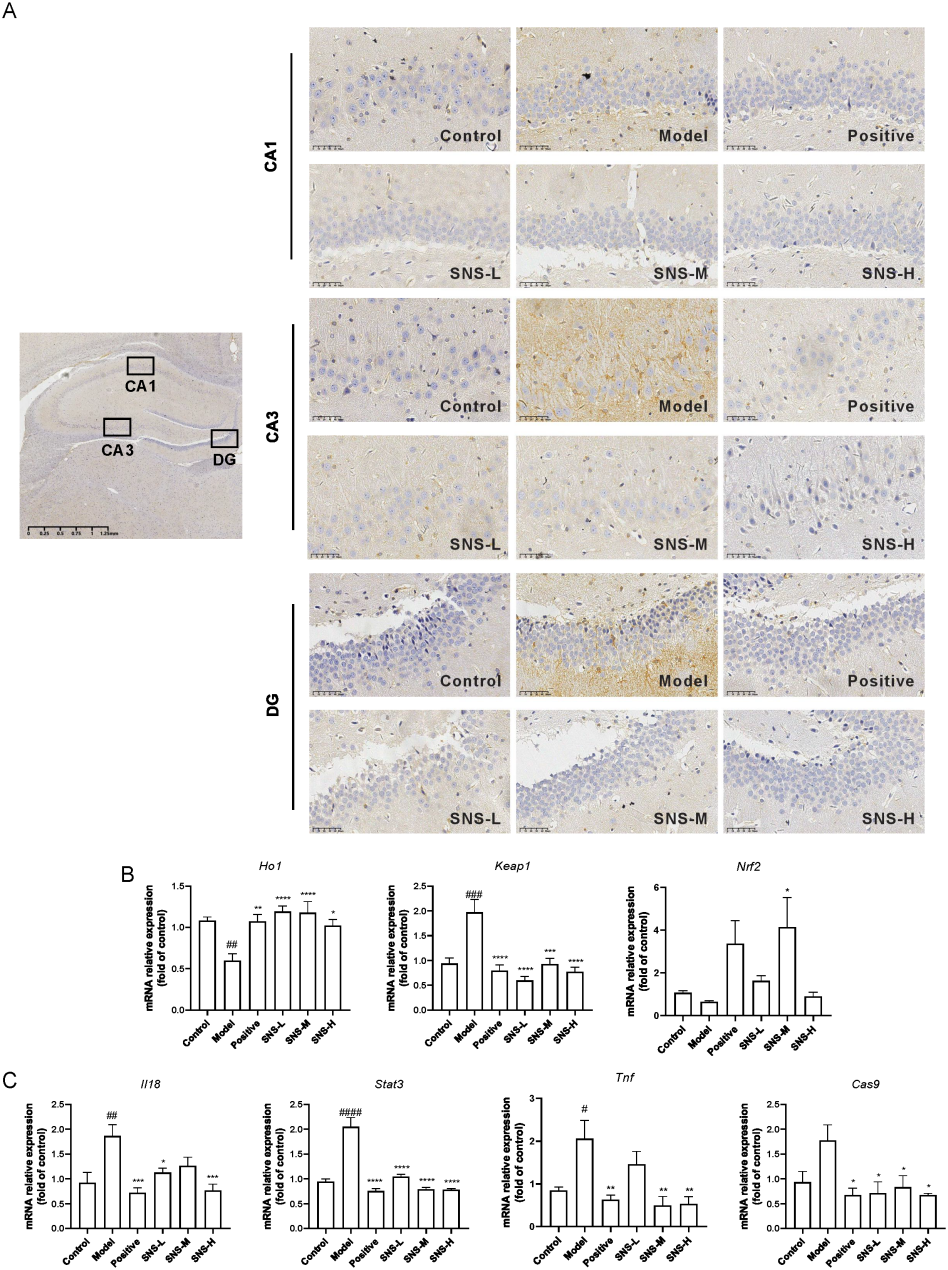
The expressions of key targets. **(A)** Immunohistochemical representative images of NFκB expressions. **(B)** The mRNA levels of *Keap1*, *Ho1* and *Nrf2.* **(C)** The mRNA levels of *Il18*, *Stat3*, *Tnf* and *Cas9.* Data were expressed as mean ± S.E.M, n=6. ^#^ *P*<0.05, ^##^ *P*<0.01, ^###^ *P*<0.005, ^####^ *P*<0.001 vs. Control group; ^*^ *P*<0.05, ^**^ *P*<0.01, ^***^ *P*<0.005, ^****^ *P*<0.001 vs. Model group.

To verify the results of network pharmacology, molecular docking and MD stimulation, the protein and mRNA expression levels of key targets in the hippocampus were detected. CA1, CA3 and DG in hippocampus are close related to depression (24), thus, the NFκB expression in those areas was detected. As shown in **Figure 6A**, high expression of NFκB was observed in CA1, CA3 and DG areas in model groups while SNS significantly reduced those expressions. Also, *Ho1* and *Nrf2* were down-regulated in Model group while Si-ni San significantly increased those expressions, and *Keap1* mRNA was up-regulated by MS+CUMS while both Si-ni San and fluoxetine were decreased this expression (**Figure 6B**). Furthermore, the inflammatory response related genes, such as *Il18*, *Stat3*, *Tnf* and *Cas9* were increased in model rats, and those in rats subjected to drug intervention were down-regulated (**Figure 6C**). Our results indicated that Si-ni San inhibited inflammatory response in hippocampus.

## 4 Discussion

Depression combined with anxiety is one of common adolescent mental diseases, and early intervention is an important link in prophylaxis and treatment (25). As is well known, CUMS is a typical cause of adolescent mental diseases, also, there is a significant correlation between emotional abuse in children and depressive symptoms (26, 27). A clinical study has shown that adolescents with a history of childhood abuse are three times more likely to suffer from depression than those without (28). These results confirm that early life adverse stress is a key risk factor and should be treated and intervened in a timely manner. The pathogenic characteristics of depression are neuroimmune abnormalities, which can be induced by unhealthy diet, lack of exercise, and long-term psychological stress, leading to an increase in inflammation and increase the risk of depression (29). Therefore, one of options for treating depression combined with anxiety is to alleviate depression- and anxiety- like behaviors by controlling neuroinflammation (30, 31). Si-ni San is an effective traditional Chinese medicine formula that has been widely used in the clinical treatment of depression (32). Previous studies have shown that Si-ni San has certain therapeutic effects in improving inflammatory response and treating inflammatory diseases, including colitis, depression, fatty liver, etc (33–35). Although our previous research also found that it can improve the expression of synaptic plasticity proteins in the hippocampus of depressed rats (12), but the specific pharmacological mechanism is not yet clear.

It was known that early adverse life stress increased mental diseases through the increased sensitivity of stress in human and animal studies (36, 37). In order to simulate that adverse experiences in early life increase the risk of depression in adult patients, this study used the MS+CUMS paradigm combined with a strategy based on network pharmacology and molecular docking to investigate the potential molecular mechanisms of Si-ni San in the treatment of adolescent depression combined with anxiety. It is worth noting that 5 active components of Si-ni San and 101 overlapping targets related to depression and anxiety were screened. Bioinformatics analysis shows that these targets are mainly related to the TNF signaling pathway, Estrogen signaling pathway and MAPK signaling pathway, indicating that the active components of Si-ni San are crucial for the pathogenesis of adolescent depression combined with anxiety.

In recent years, molecular docking has been regarded as one of the important auxiliary tools for drug design and discovery, which can predict the molecular interactions between a protein and a ligand (38). Cyclooxygenase-2 (PTGS2/COX-2), a key enzyme in the biosynthesis of prostaglandins, is closely related to biological processes such as inflammation and pain (39). It was found that prenatal activation of cyclooxygenase disrupts the formation of the blood-brain barrier, leading to lifelong brain inflammation (40), indicating that PTGS2 is an ethical contributor to neuropsychiatric disorders. Many chemical drugs and natural products exert therapeutic effects on depression by regulating COX-2 levels, through pathways such as neuroinflammation, gut microbiota, neurotransmitters, HPA axis, mitochondrial dysfunction, and hippocampal neuronal damage (41). This study aims to explore the pharmacological targets of Si-ni San by molecular docking the identified active ingredients with anti-depressant and anti-anxiety targets. Notably, quercetin, luteolin, kaempferol, nobiletin and formononetin have been screened to be crucial for the treatment of adolescent depression combined with anxiety with Si-ni San, which exhibited strong binding affinities to PTGS2. Our results are in consistent with previous reports that these active components of Si-ni San directly inhibit PTGS2 mediated inflammatory signaling (42–46). Peroxisome proliferator-activated receptor gamma (PPARγ) is a ligand activated transcription factor belonging to the nuclear receptor family, and its activity is directly regulated by the binding of steroids and thyroid hormones, vitamins, lipid metabolites and exogenous substances (47). Also, the results of MD stimulation showed that there were strong affinities between PTGS2, PPARγ and active compounds, indicating the formation of stable complexes between compounds and proteins. Studies showed that abnormal expression of PPARγ caused by diet, stress, coronary artery disease, stroke, diabetes, osteoporosis and other factors can increase the risk of depression (48, 49). Although there is no evidence to prove the direct relationship between PPARγ and neuroinflammation in patients with depression, but it is proved that its regulation of cholesterol and inflammation can improve Alzheimer’s disease by preventing nerve damage (50). Recently, study has reported that by activating PPARγ, chronic mild stress-induced inflammatory response in microglia could be blocked and the depression- and anxiety-like behaviors can be ultimately improved (51). Similarly, our results also confirmed that the top 5 active ingredients of Si-ni San is associated to PPARγ with a good combination, indicating that these components may improve neuro-inflammation by activating PPARγ signaling pathway, similar to previous studies (52–56). It was found a significant association between ESR1 polymorphism and childhood emotional disorders (57). The simultaneous absence of multiple pro-inflammatory pathways, such as NOS2, has antidepressant effects at baseline (58). Part of the components of Si-ni San can bind to NOS2 and ESR1, which may play a role in improving depression combined with anxiety through these pathways. Glycogen synthase kinase 3β (GSK-3β) is essential for long-term depression, and research has found that PSD-95, a major postsynaptic density (PSD) scaffold protein that promotes synaptic strength, could be instability induced by GSK-3β phosphorylation on T19 (59). The active ingredients of Si-ni San combined with GSK3β might regulate the expression of PSD95, which is consistent with our previous study (12).

To further validate the results of network pharmacology, we first demonstrated in animal models that Si-ni San has an improvement effect on depression- and anxiety-like behaviors caused by early stress. As expected, Si-ni San and fluoxetine have similar anti-depressant and anti-anxiety effects. The hippocampus is the most commonly studied brain region in depression research, on the one hand, the hippocampus is a part of the limbic system, forming nerve fiber connections with emotion related brain regions such as the prefrontal cortex and amygdala; on the other hand, the hippocampus contains high levels of glucocorticoid receptors and glutamate, and regulates the hypothalamic pituitary adrenal (HPA) axis, making it more susceptible to stress and depression (60). Thus, the mRNA levels of key targets associated with depression combined with anxiety in the hippocampus were detected in this study. Due to the higher oxygen consumption rate of the brain, it is more susceptible to oxidative stress damage. In severe depression and anxiety, oxidative stress and subsequent pro-inflammatory signals are important pathological mechanisms (61). Study has shown that regulating various cellular signaling pathways related to oxidative stress and inflammation plays an important role in the prevention and treatment of depression (62). The nuclear factor erythroid 2-related factor 2 (Nrf2) may play a role in inhibiting oxidative stress and related pathological processes in the antioxidant defense system, which can help improve depression (63). The Keap1-Nrf2 system is one of the pathways that resist endogenous and exogenous stress (64). By regulating the Nrf2 signaling pathway, oxidative stress and inflammatory responses can be alleviated, providing brain protection for traumatic brain injury and stress stimulation (65, 66). Our results indicated that Si-ni San could regulate the Nrf2 pathway, which might be one of the reasons why Si-ni San could improve synaptic plasticity in our previous study, as Nrf2 was involved in regulating hippocampal synaptic and functional connectivity damage in depression (67).

Finally, to evaluate the level of inflammation in the hippocampus, the expressions of inflammation related genes were detected. Interleukin-18 (IL18) was initially thought to be an inflammation induced cytokine secreted by immune cells, but recently research has focused on its non immune functions, such as its role in energy homeostasis and neural stability (68). Its high expression or genetics may increase people’s depressive response to stressful life events (69). Consistent with this, Si-ni San inhibited the expression of IL18, which also contribute to the improvement of depression combined with anxiety. Meanwhile, the TNF-STAT3 signaling axis is an inflammatory driving factor leading to blood-brain barrier (BBB) dysfunction (70), while vascular endothelial growth factor (VEGF) is also mediated (BBB) dysfunction and stress-induced depression (71). Our PCR results suggested that Si-ni San significantly inhibited TNF-STAT3 signaling, and our network pharmacology results indicated that Si-ni San is closely related to VEGFA, suggesting that Si-ni San may improve BBB dysfunction and stress through TNF-STAT3 signaling pathway and VEGF signaling pathway, which required further experimental results to verify. Caspases are involved in neuronal cell death in neurological diseases, and Caspase9 is an important indicator of cell apoptosis (72). Studies have shown that the stimulation of inflammatory factors can induce cell apoptosis by activating the NFKB signaling pathway (73), while inhibiting TNF signaling can block this process (74). Our research indicated that the expression of Caspase9 significantly inhibited by Si-ni San, consistent with the trend of TNF mRNA level. It was suggested that Si-ni San could alleviate neuroinflammation while inhibiting neuronal apoptosis, which is consistent with our previous research results (12).

## 5 Conclusion

This study investigates the role of Si-ni San in a model of depression combined with anxiety in adolescent rats, based on network pharmacology and molecular docking. This study demonstrates that adolescent anxiety- and depression-like behavior induced by MS combined CUMS can be ameliorated by Si-ni San by improved inflammation in hippocampus via targeting TNF pathway and Nrf2 pathway, helping to reveal the mechanism of Si-ni San in treating adolescent depression combined with anxiety.

## Data Availability

The original contributions presented in the study are included in the article/**Supplementary Material**. Further inquiries can be directed to the corresponding authors.
