# Supplementary material for: Network pharmacology- and molecular docking-based investigation on the mechanism of action of Si-ni San in the treatment of depression combined with anxiety and experimental verification in adolescent rats
Source: Front Psychiatry. 2024 Aug 23;15:1414242. doi: 10.3389/fpsyt.2024.1414242 (PMC11378754; doi:10.3389/fpsyt.2024.1414242)
Supplement: Supplementary file 1 [file Table1.docx]

| **Supplementary Table 1.** Active ingredients of Si-ni San screened by the TCMSP. | | | | | |
| --- | --- | --- | --- | --- | --- |
| Mol ID | Molecule Name | MW | OB (%) | DL | Source |
| MOL001918 | paeoniflorgenone | 318.35 | 87.59 | 0.37 | Baishao |
| MOL001925 | paeoniflorin_qt | 318.35 | 68.18 | 0.40 | Baishao |
| MOL001928 | albiflorin_qt | 318.35 | 66.64 | 0.33 | Baishao |
| MOL001910 | 11alpha,12alpha-epoxy-3beta-23-dihydroxy-30-norolean-20-en-28,12beta-olide | 470.71 | 64.77 | 0.38 | Baishao |
| MOL000492 | (+)-catechin | 290.29 | 54.83 | 0.24 | Baishao |
| MOL001924 | paeoniflorin | 480.51 | 53.87 | 0.79 | Baishao |
| MOL001921 | Lactiflorin | 462.49 | 49.12 | 0.80 | Baishao |
| MOL001919 | (3S,5R,8R,9R,10S,14S)-3,17-dihydroxy-4,4,8,10,14-pentamethyl-2,3,5,6,7,9-hexahydro-1H-cyclopenta[a]phenanthrene-15,16-dione | 358.52 | 43.56 | 0.53 | Baishao |
| MOL000358 | beta-sitosterol | 414.79 | 36.91 | 0.75 | Baishao |
| MOL000513 | Gallic acid | 170.13 | 31.69 | 0.04 | Baishao |
| MOL001930 | benzoyl paeoniflorin | 584.62 | 31.27 | 0.75 | Baishao |
| MOL001927 | albiflorin | 480.51 | 12.09 | 0.77 | Baishao |
| MOL005089 | Oxypaeoniflorin | 496.51 | 8.38 | 0.78 | Baishao |
| MOL001932 | Galloyl paeoniflorin | 632.62 | 3.03 | 0.42 | Baishao |
| MOL009092 | 1,2,3,4,6-O-Pentagalloylglucose | 940.72 | 3.01 | 0.21 | Baishao |
| MOL004644 | Sainfuran | 286.30 | 79.91 | 0.23 | Chaihu |
| MOL013187 | Cubebin | 356.40 | 57.13 | 0.64 | Chaihu |
| MOL004919 | METHYL CYCLOHEXANE | 98.21 | 56.20 | 0.01 | Chaihu |
| MOL000723 | 2,4-Decadienal | 152.26 | 51.03 | 0.02 | Chaihu |
| MOL004609 | Areapillin | 360.34 | 48.96 | 0.41 | Chaihu |
| MOL004628 | Octalupine | 264.41 | 47.82 | 0.28 | Chaihu |
| MOL004624 | Longikaurin A | 348.48 | 47.72 | 0.53 | Chaihu |
| MOL004653 | (+)-Anomalin | 426.50 | 46.06 | 0.66 | Chaihu |
| MOL000449 | Stigmasterol | 412.77 | 43.83 | 0.76 | Chaihu |
| MOL004718 | α-spinasterol | 412.77 | 42.98 | 0.76 | Chaihu |
| MOL001645 | Linoleyl acetate | 308.56 | 42.10 | 0.20 | Chaihu |
| MOL002776 | Baicalin | 446.39 | 40.12 | 0.75 | Chaihu |
| MOL004637 | Saikosaponin D | 781.10 | 34.39 | 0.09 | Chaihu |
| MOL004635 | saikosaponin a | 781.10 | 32.39 | 0.09 | Chaihu |
| MOL004598 | 3,5,6,7-tetramethoxy-2-(3,4,5-trimethoxyphenyl)chromone | 432.46 | 31.97 | 0.59 | Chaihu |
| MOL004648 | Troxerutin | 346.56 | 31.60 | 0.28 | Chaihu |
| MOL004702 | saikosaponin c_qt | 472.78 | 30.50 | 0.63 | Chaihu |
| MOL000490 | petunidin | 317.29 | 30.05 | 0.31 | Chaihu |
| MOL004652 | Ergosterol glucoside | 574.93 | 21.20 | 0.63 | Chaihu |
| MOL000298 | Ergosterol | 396.72 | 14.29 | 0.72 | Chaihu |
| MOL004636 | Saikosaponin B | 781.10 | 5.94 | 0.13 | Chaihu |
| MOL004701 | saikosaponin c | 943.26 | 5.12 | 0.05 | Chaihu |
| MOL010584 | Daucosterol | 590.98 | 2.72 | 0.60 | Chaihu |
| MOL002311 | Glycyrol | 366.39 | 90.78 | 0.67 | Gancao |
| MOL001789 | isoliquiritigenin | 256.27 | 85.32 | 0.15 | Gancao |
| MOL004990 | 7,2',4'-trihydroxy－5-methoxy-3－arylcoumarin | 300.28 | 83.71 | 0.27 | Gancao |
| MOL004904 | licopyranocoumarin | 384.41 | 80.36 | 0.65 | Gancao |
| MOL004891 | shinpterocarpin | 322.38 | 80.30 | 0.73 | Gancao |
| MOL005017 | Phaseol | 336.36 | 78.77 | 0.58 | Gancao |
| MOL004841 | Licochalcone B | 286.30 | 76.76 | 0.19 | Gancao |
| MOL004810 | glyasperin F | 354.38 | 75.84 | 0.54 | Gancao |
| MOL001484 | Inermine | 284.28 | 75.18 | 0.54 | Gancao |
| MOL000500 | Vestitol | 272.32 | 74.66 | 0.21 | Gancao |
| MOL005007 | Glyasperins M | 368.41 | 72.67 | 0.59 | Gancao |
| MOL004941 | (2R)-7-hydroxy-2-(4-hydroxyphenyl)chroman-4-one | 256.27 | 71.12 | 0.18 | Gancao |
| MOL004959 | 1-Methoxyphaseollidin | 354.43 | 69.98 | 0.64 | Gancao |
| MOL000392 | formononetin | 268.28 | 69.67 | 0.21 | Gancao |
| MOL004863 | 3-(3,4-dihydroxyphenyl)-5,7-dihydroxy-8-(3-methylbut-2-enyl)chromone | 354.38 | 66.37 | 0.41 | Gancao |
| MOL004903 | liquiritin | 418.43 | 65.69 | 0.74 | Gancao |
| MOL004808 | glyasperin B | 370.43 | 65.22 | 0.44 | Gancao |
| MOL002844 | Pinocembrin | 256.27 | 64.72 | 0.18 | Gancao |
| MOL004829 | Glepidotin B | 340.40 | 64.46 | 0.34 | Gancao |
| MOL004855 | Licoricone | 382.44 | 63.58 | 0.47 | Gancao |
| MOL004914 | 1,3-dihydroxy-8,9-dimethoxy-6-benzofurano[3,2-c]chromenone | 328.29 | 62.90 | 0.53 | Gancao |
| MOL004835 | Glypallichalcone | 284.33 | 61.60 | 0.19 | Gancao |
| MOL004907 | Glyzaglabrin | 298.26 | 61.07 | 0.35 | Gancao |
| MOL005000 | Gancaonin G | 352.41 | 60.44 | 0.39 | Gancao |
| MOL004824 | (2S)-6-(2,4-dihydroxyphenyl)-2-(2-hydroxypropan-2-yl)-4-methoxy-2,3-dihydrofuro[3,2-g]chromen-7-one | 384.41 | 60.25 | 0.63 | Gancao |
| MOL004849 | 3-(2,4-dihydroxyphenyl)-8-(1,1-dimethylprop-2-enyl)-7-hydroxy-5-methoxy-coumarin | 368.41 | 59.62 | 0.43 | Gancao |
| MOL005003 | Licoagrocarpin | 338.43 | 58.81 | 0.58 | Gancao |
| MOL004838 | 8-(6-hydroxy-2-benzofuranyl)-2,2-dimethyl-5-chromenol | 308.35 | 58.44 | 0.38 | Gancao |
| MOL005012 | Licoagroisoflavone | 336.36 | 57.28 | 0.49 | Gancao |
| MOL005018 | Xambioona | 388.49 | 54.85 | 0.87 | Gancao |
| MOL005020 | dehydroglyasperins C | 340.40 | 53.82 | 0.37 | Gancao |
| MOL004993 | 8-prenylated eriodictyol | 356.40 | 53.79 | 0.40 | Gancao |
| MOL004908 | Glabridin | 324.40 | 53.25 | 0.47 | Gancao |
| MOL004910 | Glabranin | 324.40 | 52.90 | 0.31 | Gancao |
| MOL004879 | Glycyrin | 382.44 | 52.61 | 0.47 | Gancao |
| MOL004912 | Glabrone | 336.36 | 52.51 | 0.50 | Gancao |
| MOL004885 | licoisoflavanone | 354.38 | 52.47 | 0.54 | Gancao |
| MOL003656 | Lupiwighteone | 338.38 | 51.64 | 0.37 | Gancao |
| MOL004856 | Gancaonin A | 352.41 | 51.08 | 0.40 | Gancao |
| MOL000239 | Jaranol | 314.31 | 50.83 | 0.29 | Gancao |
| MOL004820 | kanzonols W | 336.36 | 50.48 | 0.52 | Gancao |
| MOL005001 | Gancaonin H | 420.49 | 50.10 | 0.78 | Gancao |
| MOL005016 | Odoratin | 314.31 | 49.95 | 0.30 | Gancao |
| MOL004848 | licochalcone G | 354.43 | 49.25 | 0.32 | Gancao |
| MOL002565 | Medicarpin | 270.30 | 49.22 | 0.34 | Gancao |
| MOL004857 | Gancaonin B | 368.41 | 48.79 | 0.45 | Gancao |
| MOL004827 | Semilicoisoflavone B | 352.36 | 48.78 | 0.55 | Gancao |
| MOL004913 | 1,3-dihydroxy-9-methoxy-6-benzofurano[3,2-c]chromenone | 298.26 | 48.14 | 0.43 | Gancao |
| MOL000417 | Calycosin | 284.28 | 47.75 | 0.24 | Gancao |
| MOL004961 | Quercetin der. | 330.31 | 46.45 | 0.33 | Gancao |
| MOL004898 | (E)-3-[3,4-dihydroxy-5-(3-methylbut-2-enyl)phenyl]-1-(2,4-dihydroxyphenyl)prop-2-en-1-one | 340.40 | 46.27 | 0.31 | Gancao |
| MOL004911 | Glabrene | 322.38 | 46.27 | 0.44 | Gancao |
| MOL004974 | 3'-Methoxyglabridin | 354.43 | 46.16 | 0.57 | Gancao |
| MOL004811 | Glyasperin C | 356.45 | 45.56 | 0.40 | Gancao |
| MOL004949 | Isolicoflavonol | 354.38 | 45.17 | 0.42 | Gancao |
| MOL000432 | Linoleic acid | 278.48 | 45.01 | 0.15 | Gancao |
| MOL004828 | Glepidotin A | 338.38 | 44.72 | 0.35 | Gancao |
| MOL004948 | Isoglycyrol | 366.39 | 44.70 | 0.84 | Gancao |
| MOL004866 | 2-(3,4-dihydroxyphenyl)-5,7-dihydroxy-6-(3-methylbut-2-enyl)chromone | 354.38 | 44.15 | 0.41 | Gancao |
| MOL004966 | 3'-Hydroxy-4'-O-Methylglabridin | 354.43 | 43.71 | 0.57 | Gancao |
| MOL004915 | Eurycarpin A | 338.38 | 43.28 | 0.37 | Gancao |
| MOL003896 | 7-Methoxy-2-methyl isoflavone | 266.31 | 42.56 | 0.20 | Gancao |
| MOL000131 | Linolelaidic acid | 280.50 | 41.90 | 0.14 | Gancao |
| MOL004883 | Licoisoflavone | 354.38 | 41.61 | 0.42 | Gancao |
| MOL005008 | Glycyrrhiza flavonol A | 370.38 | 41.28 | 0.60 | Gancao |
| MOL005013 | 18α-hydroxyglycyrrhetic acid | 486.76 | 41.16 | 0.71 | Gancao |
| MOL004924 | (-)-Medicocarpin | 432.46 | 40.99 | 0.95 | Gancao |
| MOL000497 | licochalcone a | 338.43 | 40.79 | 0.29 | Gancao |
| MOL004980 | Inflacoumarin A | 322.38 | 39.71 | 0.33 | Gancao |
| MOL004815 | (E)-1-(2,4-dihydroxyphenyl)-3-(2,2-dimethylchromen-6-yl)prop-2-en-1-one | 322.38 | 39.62 | 0.35 | Gancao |
| MOL004989 | 6-prenylated eriodictyol | 356.40 | 39.22 | 0.41 | Gancao |
| MOL004884 | Licoisoflavone B | 352.36 | 38.93 | 0.55 | Gancao |
| MOL004991 | 7-Acetoxy-2-methylisoflavone | 294.32 | 38.92 | 0.26 | Gancao |
| MOL004957 | HMO | 268.28 | 38.37 | 0.21 | Gancao |
| MOL004917 | glycyroside | 562.57 | 37.25 | 0.79 | Gancao |
| MOL004945 | (2S)-7-hydroxy-2-(4-hydroxyphenyl)-8-(3-methylbut-2-enyl)chroman-4-one | 324.40 | 36.57 | 0.32 | Gancao |
| MOL004978 | 2-[(3R)-8,8-dimethyl-3,4-dihydro-2H-pyrano[6,5-f]chromen-3-yl]-5-methoxyphenol | 338.43 | 36.21 | 0.52 | Gancao |
| MOL004935 | Sigmoidin-B | 356.40 | 34.88 | 0.41 | Gancao |
| MOL004905 | 3,22-Dihydroxy-11-oxo-delta(12)-oleanene-27-alpha-methoxycarbonyl-29-oic acid | 512.75 | 34.32 | 0.55 | Gancao |
| MOL004882 | Licocoumarone | 340.40 | 33.21 | 0.36 | Gancao |
| MOL004860 | licorice glycoside E | 693.71 | 32.89 | 0.27 | Gancao |
| MOL001792 | DFV | 256.27 | 32.76 | 0.18 | Gancao |
| MOL004988 | Kanzonol F | 420.54 | 32.47 | 0.89 | Gancao |
| MOL004833 | Phaseolinisoflavan | 324.40 | 32.01 | 0.45 | Gancao |
| MOL004814 | Isotrifoliol | 298.26 | 31.94 | 0.42 | Gancao |
| MOL004805 | (2S)-2-[4-hydroxy-3-(3-methylbut-2-enyl)phenyl]-8,8-dimethyl-2,3-dihydropyrano[2,3-f]chromen-4-one | 390.51 | 31.79 | 0.72 | Gancao |
| MOL004985 | icos-5-enoic acid | 310.58 | 30.70 | 0.20 | Gancao |
| MOL004996 | gadelaidic acid | 310.58 | 30.70 | 0.20 | Gancao |
| MOL004864 | 5,7-dihydroxy-3-(4-methoxyphenyl)-8-(3-methylbut-2-enyl)chromone | 352.41 | 30.49 | 0.41 | Gancao |
| MOL004806 | euchrenone | 406.56 | 30.29 | 0.57 | Gancao |
| MOL004876 | Glycyrrhizic acid | 823.04 | 19.62 | 0.11 | Gancao |
| MOL004804 | Glycyrrhetinic acid | 470.76 | 17.65 | 74.60 | Gancao |
| MOL013353 | synephrine | 167.23 | 79.00 | 0.04 | Zhishi |
| MOL007561 | N-Methyltyramine | 151.23 | 75.52 | 0.03 | Zhishi |
| MOL013433 | prangenin hydrate | 304.32 | 72.63 | 0.29 | Zhishi |
| MOL001798 | neohesperidin_qt | 302.30 | 71.17 | 0.27 | Zhishi |
| MOL002341 | Hesperetin | 302.30 | 70.31 | 0.27 | Zhishi |
| MOL013435 | poncimarin | 330.41 | 63.62 | 0.35 | Zhishi |
| MOL013436 | isoponcimarin | 330.41 | 63.28 | 0.31 | Zhishi |
| MOL005828 | nobiletin | 402.43 | 61.67 | 0.52 | Zhishi |
| MOL004358 | linalool | 140.25 | 58.18 | 0.02 | Zhishi |
| MOL013277 | Isosinensetin | 372.40 | 51.15 | 0.44 | Zhishi |
| MOL009053 | 4-[(2S,3R)-5-[(E)-3-hydroxyprop-1-enyl]-7-methoxy-3-methylol-2,3-dihydrobenzofuran-2-yl]-2-methoxy-phenol | 358.42 | 50.76 | 0.39 | Zhishi |
| MOL001803 | Sinensetin | 372.40 | 50.56 | 0.45 | Zhishi |
| MOL013443 | isolimonic acid | 639.02 | 48.86 | 0.18 | Zhishi |
| MOL005100 | 5,7-dihydroxy-2-(3-hydroxy-4-methoxyphenyl)chroman-4-one | 302.30 | 47.74 | 0.27 | Zhishi |
| MOL007879 | Tetramethoxyluteolin | 342.37 | 43.68 | 0.37 | Zhishi |
| MOL013430 | Prangenin | 286.30 | 43.60 | 0.29 | Zhishi |
| MOL013352 | Obacunone | 454.56 | 43.29 | 0.77 | Zhishi |
| MOL002914 | Eriodyctiol (flavanone) | 288.27 | 41.35 | 0.24 | Zhishi |
| MOL013428 | isosakuranetin-7-rutinoside | 594.62 | 41.24 | 0.72 | Zhishi |
| MOL013440 | citrusin B | 568.63 | 40.80 | 0.71 | Zhishi |
| MOL000023 | limonene | 136.26 | 39.84 | 0.02 | Zhishi |
| MOL013279 | 5,7,4'-Trimethylapigenin | 312.34 | 39.83 | 0.30 | Zhishi |
| MOL005849 | didymin | 286.30 | 38.55 | 0.24 | Zhishi |
| MOL013276 | poncirin | 594.62 | 36.55 | 0.74 | Zhishi |
| MOL001941 | Ammidin | 270.30 | 34.55 | 0.22 | Zhishi |
| MOL013437 | 6-Methoxy aurapten | 328.44 | 31.24 | 0.30 | Zhishi |
| MOL007930 | hesperidin | 610.62 | 13.33 | 0.67 | Zhishi |
| MOL013383 | Neohesperidin | 610.62 | 11.57 | 0.69 | Zhishi |
| MOL013336 | Narirutin | 580.59 | 8.15 | 0.75 | Zhishi |
| MOL005812 | naringin | 580.59 | 6.92 | 0.78 | Zhishi |
| MOL000010 | Rhoifolin | 578.57 | 6.68 | 0.77 | Zhishi |
| MOL009088 | Neoeriocitrin | 596.59 | 3.93 | 0.73 | Zhishi |
| MOL000768 | Lonicerin | 594.57 | 3.84 | 0.73 | Zhishi |
| MOL000211 | Mairin | 456.78 | 55.38 | 0.78 | Baishao，Gancao |
| MOL000422 | kaempferol | 286.25 | 41.88 | 0.24 | Baishao，Chaihu，Gancao |
| MOL000359 | sitosterol | 414.79 | 36.91 | 0.75 | Baishao，Chaihu，Zhishi |
| MOL000354 | isorhamnetin | 316.28 | 49.60 | 0.31 | Chaihu，Gancao |
| MOL000098 | quercetin | 302.25 | 46.43 | 0.28 | Chaihu，Gancao |
| MOL000006 | Luteolin | 286.25 | 36.16 | 0.25 | Chaihu，Zhishi |
| MOL004328 | naringenin | 272.27 | 59.29 | 0.21 | Gancao，Zhishi |
| Note: The OB% and DL values of some compounds do not meet the requirements, but they have been reported in the literature, so they were included in the active compound component library | | | | | |

| **MOL ID** | **Molecule Name** | **MW** | **OB(%) (%)** | **DL** |
| --- | --- | --- | --- | --- |
| MOL000098 | quercetin | 302.25 | 46.43 | 0.28 |
| MOL000006 | luteolin | 286.25 | 36.16 | 0.25 |
| MOL000422 | kaempferol | 286.25 | 41.88 | 0.24 |
| MOL005828 | nobiletin | 402.43 | 61.67 | 0.52 |
| MOL000392 | formononetin | 268.28 | 69.67 | 0.21 |

**Supplementary Table 2.** Active ingredients of Si-ni San screened by the TCMSP.

**Supplementary Table 3.** The affinity of Si-ni San active ingredients with PTSG2 and PPARγ.

| **Molecule Name** | **Protein (Uniprot_ID)** | **PDB_ID** | **Binding free energy**  **(kcal·mol^-1^)** | **hydrogen bond residue** **account** | **van der Waals residue account** | **Other interaction residue account** |
| --- | --- | --- | --- | --- | --- | --- |
|  |  |  |  |  |  |  |
| quercetin | PTGS2 (P03372) | 5f1q | -7.49 | 3 | 9 | 1 |
| luteolin |  | 5f1q | -7.12 | 4 | 6 | 1 |
| kaempferol |  | 5f1q | -7.19 | 5 | 5 | 2 |
| quercetin | PPARγ (P37231) | 3et3 | -7.27 | 5 | 11 | 4 |
| luteolin |  | 3et3 | -7.06 | 3 | 5 | 4 |

**Supplementary Table 4.**  The binding energy by MMGBSA (kJ/mol).

| Type | PPARG-Luteolin | PPARG-Quercetin | PTGS2-Kaemoferol | PTGS2-Luteolin | PTGS2-Quercetin |
| --- | --- | --- | --- | --- | --- |
| EVDW | -169.898+/-10.586 | -154.955+/-7.53 | -151.15+/-8.2 | -169.571+/-11.2 | -159.671+/-7.632 |
| EELE | -38.85+/-7.74 | -46.102+/-5.95 | -55.388+/-7.801 | -38.715+/-14.885 | -23.131+/-4.359 |
| EGB | 146.363+/-5.306 | 162.99+/-6.447 | 163.175+/-7.029 | 129.154+/-11.574 | 134.31+/-8.032 |
| ESA | -15.288+/-0.738 | -16.621+/-0.66 | -14.37+/-0.473 | -14.576+/-0.606 | -13.547+/-0.756 |
| Gbinding energy | -77.673+/-9.21 | -54.688+/-9.36 | -57.733+/-8.967 | -93.708+/-9.953 | -62.039+/-7.464 |

*E_VDW_*: van der Waals energy

*E_ELE_*: eletrostatic energy

*E_GB_*: polar contribution to solvation

*E_SA_*: non-polar contribution to solvation
