# Supplementary figures and images for: Network pharmacology- and molecular docking-based investigation on the mechanism of action of Si-ni San in the treatment of depression combined with anxiety and experimental verification in adolescent rats
Source: Front Psychiatry. 2024 Aug 23;15:1414242. doi: 10.3389/fpsyt.2024.1414242 (PMC11378754; doi:10.3389/fpsyt.2024.1414242)

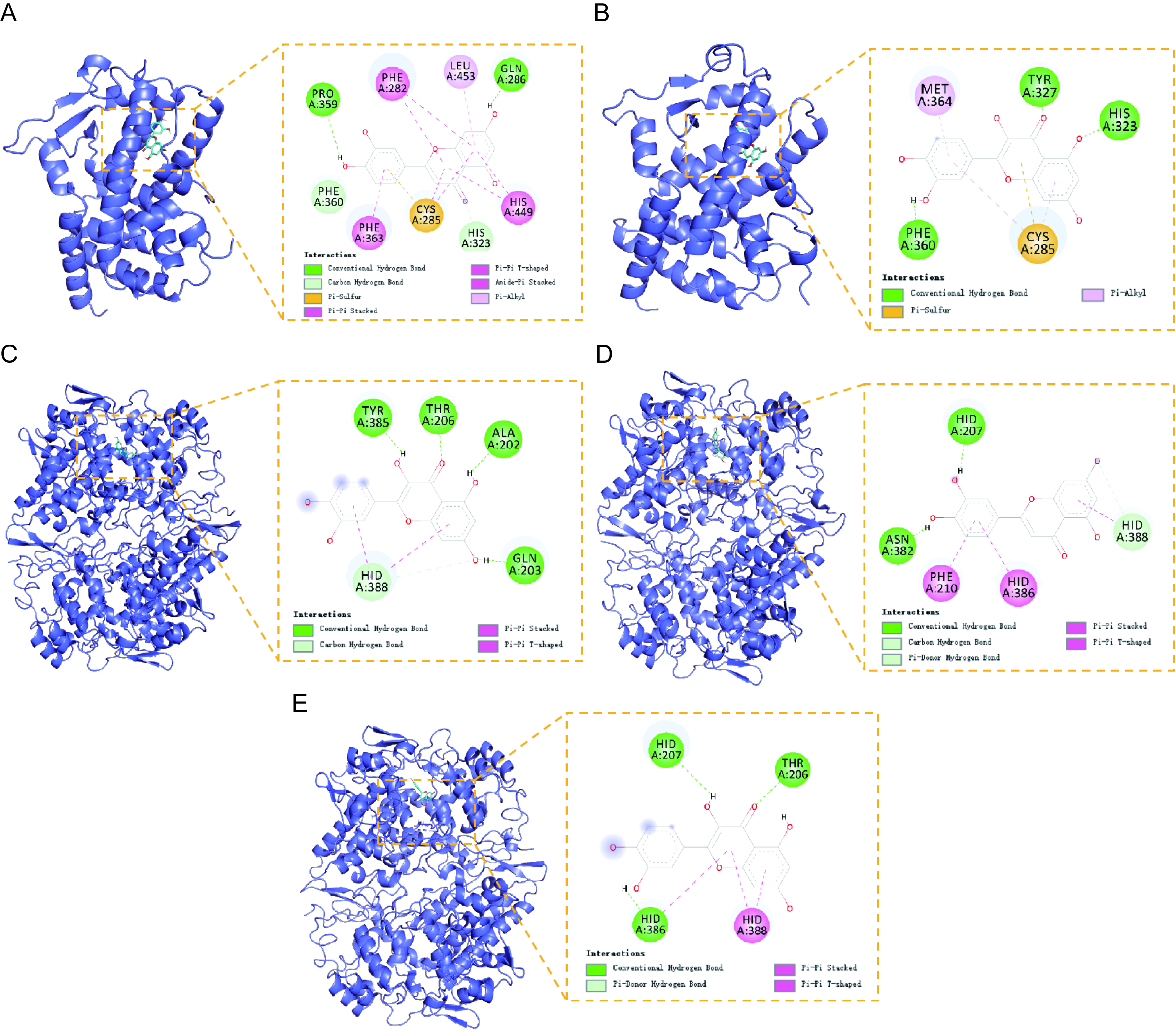

Supplement: Supplementary file 2 [file Image1.tif]
